# Subjective judgments of rhythmic complexity in Parkinson’s disease: Higher baseline, preserved relative ability, and modulated by tempo

**DOI:** 10.1371/journal.pone.0221752

**Published:** 2019-09-03

**Authors:** Kjetil Vikene, Geir Olve Skeie, Karsten Specht

**Affiliations:** 1 Department of Biological and Medical Psychology, University of Bergen, Bergen, Norway; 2 Mohn Medical Imaging and Visualization Centre, Haukeland University Hospital, Bergen, Norway; 3 Department of Neurology, Haukeland University Hospital, Bergen, Norway; 4 The Grieg Academy - Department of Music, University of Bergen, Norway; 5 Department of Education, The Arctic University of Norway, Tromsø, Norway; IMT Atlantique, FRANCE

## Abstract

Previous research has demonstrated that people with Parkinson’s disease (PD) have difficulties with the perceptual discrimination of rhythms, relative to healthy controls. It is not however clear if this applies only to simpler rhythms (a so called “beat-based” deficit), or if it is a more generalized deficit that also applies to more complex rhythms. Further insight into how people with PD process and perceive rhythm can refine our understanding of the well known problems of temporal processing in the disease. In this study, we wanted to move beyond simple/complex-dichotomy in previous studies, and further investigate the effect of tempo on the perception of musical rhythms. To this end, we constructed ten musical rhythms with a varied degree of complexity across three different tempi. Nineteen people with PD and 19 healthy controls part-took in an internet based listening survey and rated 10 different musical rhythms for complexity and likeability. In what we believe is the first study to do so, we asked for the participants *subjective* ratings of individual rhythms and not their capacity to directly compare or discriminate between them. We found an overall between-group difference in complexity judgments that was modulated by tempo, but not level of complexity. People with PD rated all rhythms as more complex across tempi, with significant group differences in complexity ratings at 120 and 150bpm, but not at 90bpm. Our analysis found a uniform elevated baseline for complexity judgments in the PD-group, and a strong association between the two groups’ rank-ordering the rhythms for complexity. This indicates a preserved ability to discriminate between relative levels of complexity. Finally, the two groups did not significantly differ in their subjective scoring of likeability, demonstrating a dissimilarity between judgment of complexity and judgment of likeability between the two groups. This indicates different cognitive operations for the two types of judgment, and we speculate that Parkinson’s disease affects judgment of complexity but not judgment of likeability.

## Introduction

In Parkinson’s disease (PD), death of dopaminergic neurons in the substantia nigra pars compacta in the basal ganglia disrupts several subcortico-cortical loops in motor, associative, and limbic circuitry [1–3]. This leads to motor and non-motor symptoms that increase their severity with disease progression [4, 5]. While some motor symptoms in PD, such as bradykinesia and tremor, are relatively dopamine responsive [5, 6] other gait-specific symptoms, such as postural instability and balance problems [7], are relatively unresponsive to pharmacological treatment [8, 9]. Cognitive impairments are well documented in PD [10, 11], so is the effect of dopaminergic medication on cognitive functions [12]. The intimate relationship between general cognitive functioning and timing deficits in PD [13, 14] has made rhythm processing increasingly more central in the study of cognition in PD [15].

Pathologically, key brain areas and networks involved in rhythm processing in healthy subjects are found to be dysfunctional in PD. Imaging studies of rhythm processing in healthy subjects have found a crucial role for the basal ganglia in detection of auditory sequences, beat generation and beat prediction [16–18]. Involvement of cerebellar [19–22], cortical motor [23–25] and prefrontal areas [17, 26] in rhythm processing tasks have been demonstrated. These are areas directly or indirectly connected to the basal ganglia-circuitries affected in PD, where abnormal activations in cerebellar [27–29], cortical motor [29] and prefrontal areas [30] have been shown. In a recent study we have shown widespread differences in brain activation during rhythm processing in PD, including cortical areas in parietal, auditory, motor, prefrontal, cingulate and in the basal ganglia [31]. PD pathology thus affects many areas and networks associated with rhythm processing on several levels. It might therefore seem as paradoxical that while many people with PD have problems with voluntarily self-paced movement, they can synchronize to external auditory cues [32]. This effect is often explained as entrainment [33] to an external rhythmic stimuli by an intrinsic timekeeper capacity, which is disrupted in PD [34], possibly through the activation of compensatory circuitries [35]. An increasing number of studies has shown how cue-based music and rhythm therapies has positive effects on gait-symptoms [34, 36–39]. Interplay between common mechanisms in cognition and rhythm entrainment has been explored to investigate the potential of cue-based therapies to improve non-motor, cognitive functioning [35, 40, 41], i.e., that symptom reduction in one domain can benefit others.

At the core of many studies on rhythm processing is the question of differential processing of simple and complex rhythms, with imaging and behavioral studies using three levels of complexity: metric simple, metric complex and non-metric rhythms [23–25, 42, 43]. One study on rhythm processing in PD, found that people with PD are impaired in discriminating between strongly metric (isochronous or simple) rhythms relative to healthy controls, coining this as a “beat-based deficit” [44]. In discrimination of complex and non-metrical rhythms, people with PD were comparable to healthy controls [44]. The strictly metric (simple rhythms) condition of this study was recently replicated as part of another study [15]. A follow up [45] of the original study [44], using a subset of the original stimuli, with only simple/complex rhythms, testing for the effect of being on/off L-DOPA-medication on discrimination abilities, failed however to reproduce the “beat-based deficit” in the original study [44],—i.e., people with PD did not have significantly greater problems discriminating simple compared to complex rhythms, relative to controls. No interaction between group, session (ON/OFF medication) and metricality (simple/complex) were reported, and only a *general* discrimination deficit in people with PD relative to controls was found (main effect of group *p* = .0.001), irrespective then of metrical complexity [45].

This conflicting evidence in the literature motivated the current study, and the aim was to move beyond the dichotomy of simple/complex [45]. To this end we chose a model from the literature (Pressing, 1999) to construct the set of stimuli with varied complexity across 10 rhythms (see Method section for details) with the aim to compare the rank-order (and range-normalized ratings) of complexity judgments between the PD-group and the healthy control group. A pool of stimuli with more varied rhythmic complexity would allow for a more fine-grained examination of the ability of people with PD to judge complexity along a spectrum, i.e., to investigate if there is a certain point along this spectrum where complexity becomes problematic for people with PD or whether the difficulties in rhythm processing is a general deficit, independent of level of complexity.

Another important finding in the study mentioned [45] was the finding that no significant difference was found between people with PD and controls in the Beat Alignment Test (BAT), where participants are to judge if an added a sequence of regular tones overlaid on clips of “real” musical excerpts, are aligned or misaligned with the musical beat of the music. The authors speculate that the richness of real music offer more cues, facilitating beat perception in people with PD, a statement which also find support in studies showing how acoustic components such as “fullness”, “brightness” and “timbral complexity” activate larger brain-networks (outside the basal ganglia), including cerebellar areas [46]. One study speculates that the activation of a compensatory cerebellar–thalamocortical network during musically cued gait training might explain the positive effect of auditory cues in therapy in PD [35], compensating for the damaged striato-thalamo-cortical network, hypothesized to be involved in relative, beat-based timing [19]. A claim that more musical stimuli should have positive therapeutic benefits, also finds support in studies claiming that ecological valid auditory stimuli is beneficiary for movement facilitation in PD [47]. For the stimuli constructed for the current study we added ecological validity by using samples of real drum sounds, simple piano-chords to mark the start of the rhythmic patterns, as well as synthesized bass sounds added to the beat sequences, with the intent to study beat-perception differences in *musical* rhythms, as compared to sequence perception of sinusoid synthesized sounds. It is however important to note that in our stimuli only the rhythmic structure were manipulated; all other (intensities, timbre) were the same across the stimuli. In line with previous research, we hypothesized that using more ecological valid stimuli would make the task easier for people with PD, thereby potentially making differences in rhythm perception judgment smaller or even disappear. If differences were still to be found, these would speak to a “real” difference in rhythm perception abilities in PD that could not fully be compensated though the use of real music.

We hasten to add that the aim of the current study is not to discuss therapy, but to address the question of *subjective* judgment of complexity in rhythms in PD, something that has not been investigated in PD previously. Rhythm processing deficits in PD does not necessarily translate into self-reported, subjective judgments, since dysfunction at one system level (dopamine depletion in the basal ganglia) does not necessarily show itself as impairment on another system level (subjective judgment). Nevertheless, measuring self-reported judgment of complexity in rhythm will potentially show if and how rhythm-processing impairments manifests on a phenomenological level and lend detail to our understanding of the specificity of the problems of temporal processing in PD [13, 48].

In this context we were also interested in investigating was the effect of *tempo* in rhythm processing in PD. The two studies referred to above (bpm calculated on the millisecond measures stated in the articles) used tempi between 111 to 136bpm (in approx. 5bpm steps) [44] and 102, 129, and 150bpm [45] respectively. These tempi are interesting in lieu of rhythmic auditory stimulation studies performed on people with PD, where studies have found that a rhythmic auditory cues at 100% compared to a patients normal gait cadence was beneficiary to stride length and swing [38, 39, 49], velocity and cadence [49]. In these studies, natural gait cadence was found to be, on average, 111, 107 and 114bpm respectively, and in the two first of these studies, an increase to 110% of natural gait tempi (122bpm and 111bpm respectively) also significantly reduced the variability in gait. This indicates that the tempo of auditory stimuli has manifest effects on movement in PD, and we were interested in investigating whether tempo also had an effect on the perception of rhythmic complexity in people with PD.

We therefore distributed our stimuli across three different tempi to investigate whether there was an effect. The three tempi chosen were 90bpm, 120bpm and 150bpm. The first tempo significantly slower than those found for natural gait, the second an average of the three studies mentioned above (at 110%), and the third significantly faster than natural gait. In addition, a tempo at 120bpm is seen as a general tempo of human locomotion [50] and salient when walking to music [51]. As a strong auditory-motor area coupling is a dominant explanatory model for beat perception [16, 17, 52, 53], and the slightly increased tempo of 110% compared to natural gait was found to decrease movement problems in PD, we hypothesized that stimuli played at this tempo would be experienced as less complex than the slower and faster tempi, in both groups.

Perception of complexity has been found to be influenced by the listeners preference of music, with many studies showing that the level of perceived complexity is related to preference in an inverted U-curve (or Wundt-curve), with listeners preferring medium complexities to more simple or more complex rhythms [54], [55, 56]. Research into the relationship between rhythmic complexity and a feeling of “wanting to move” (i.e., dance) to music has shown similar results [57]. In this latter study, the stimuli used were at 120bpm, a tempo that is close to what another study indicates as an “optimal tempo for groove”, i.e., the feeling that induces body movement [58]. In sum, studies such as these point to a complex relationship between subjective preference, rhythmic complexities and body movements.

Studies on musical preference in relation to rhythmic cued or music therapies in PD is limited, and in relation to processing and perception (or as in our case subjective judgement) scarce. One study on PD suggested using self-selected (preferred) music did not give beneficiary effects in therapy [59], while another found positive effects of taking into consideration tempo-to-cadence matched songs, and preference and listening habits [49]. While simple, isochronous pulsed or beat-based rhythmic stimuli have been found to be very effective in therapeutic settings [60], newer research points to a combination of cue-continuity (simplicity) and action-relevance (i.e., ecologic valid sounds such as music [47]) to be a more successful combination than any one factor alone [61].

It seems logical that the relationship between preference and rhythmic complexity should play an important role in rhythm processing in PD. As a proxy or operationalization for preference, we therefore asked the participants also to rate the different stimuli for likeability. Through this, we wanted to investigate whether the relationship between likeability and rhythmic complexity was different in people with PD compared to controls. We speculated that a judgment of likeability constituted a cognitively different operation than judgment of complexity, since it does not involve a judgment of a specific temporal relationship of qualities *within* the stimuli itself (i.e., a cognitive “analysis” of a particular feature, complexity), but rather an overall more intuitive or emotional response. As a somewhat open question, we were therefore interested in investigating whether an inverted U-curve relationship between complexity and likeability would differ between the two groups. We hypothesized that if the subjective judgment of complexity in rhythm is a more general deficit in PD (independent of level of complexity) and the subjective judgment of likeability is unaffected by the pathology, a possible U-curve for the relationship between the two judgments would have the same shape, but, in the case of PD, displaced on the axis of complexity.

As with the ratings of complexity, we additionally wanted to investigate whether tempo affected ratings of likeability differently in people with PD compared to controls. Previous research into the effect of tempo on preference from children [62] to college students [63], and into the emotional impact of tempo [64] (the latter study using the three tempos 90, 120, 150bpm), all hint to increased preference and in general more positive emotional valence as tempo increases, and we therefore expected to find similar results in our study, i.e., that the higher the tempo, the higher the likeability-ratings would be, in both groups.

Finally, we wanted to briefly to address the relationship between clinical scores, cognitive abilities and rhythm processing. One study found a correlation between UPDRS-III scores and rhythm discrimination abilities [45], while another study indicate that rhythmic processing in Parkinson’s disease can be used as predictors for other cognitive operations, particularly for working memory operations [15]. In addition to the UPDRS-III clinical scores and time since diagnosis, we therefore included a small neuropsychological test battery. Since the task of judging complexity of the individual rhythms at some level must involve a covert comparison between them, the ability to encode and retain recently presented items could also affect the ability of such a covert comparison. One study indicates a separate working memory system for rhythm [65], overlapping with areas affected by PD. Of particular interest to us therefore, are findings on deficits in working memory (WM) in PD [66, 67], indicating that working memory deficits in PD are predominantly in the encoding stage [68]. Indeed, working memory networks have phasic dynamics that develop differently for time-dependent parts for encoding, maintenance and retrieval stages [69], also in auditory working memory [70] and during time perception [71]. Time-dependent changes in WM networks might in turn be modulated by stimuli-related cognitive load [72], and can furthermore be related to large-scale network dynamics, as there are indications that working memory networks de-couples from both default mode network and executive network during maintenance, possibly to avoid or minimize any external or internal distraction [69]. De-coupling of dopaminergic circuits in the basal ganglia from other networks during prolonged listening to rhythmic music [73], indicate that establishing stable temporal structures involves self-reinforcing, and perhaps rhythm specific, WM network mechanisms. Interactions between frontal areas and the basal ganglia play a crucial role in working memory [74, 75], and the temporal dynamics of basal ganglia activity during WM operations [76] shows phasic dysfunction in PD [77], and distinct phasic differences in brain activity patterns in PD during early stages of rhythm processing include differences in pre/motor, auditory, prefrontal, inferior frontal and basal ganglia areas [31], areas that overlap with WM circuitries.

A simple measure of memory and learning abilities is the California Verbal Learning Test-II (CVLT-II) and this test was administered to check for correlations between complexity ratings and verbal memory, as an indirect measure of general auditory working memory and learning impairment [68] (i.e., encoding). The test was chosen because it has previously been used in PD [78] and is also part of the standard PD assessment at the hospital where this study was undertaken. A Stroop-test [79] was administered to test for correlations with set-shifting abilities as a measure of attention and executive function [80].

## Methods and material

### Participants

Twenty-three volunteers with PD were recruited with the help of the National Parkinson’s Association in Norway. The results of three participants were removed due to problems performing the online test. Twenty healthy controls were recruited, and matched for age, sex, education level, as well as for musical expertise when possible. In two cases however female HCs were matched to male people with PD. This skewed the matching of the two groups, and we have taken this into consideration in the analyses by adding sex as a between-subject factor. Additionally one female PD and one female HC were removed when it was discovered they had not used the required time to perform the test (i.e., rushing through the test and not listening to the complete stimuli). Thus, 19 people with PD and 19 healthy controls were included in the analysis. For all participants, a minimum Mini Mental Status (MMS) [81] score of 24 was set, to exclude people with cognitive impairment indicative of dementia in both groups. All people with PD were rated with the Unified Parkinson’s disease rating scale, part III (UPDRS-III) [82]. All people with PD were in medication regimens (L-DOPA, D2-antagonists, MAO-inhibitors and or COMT-inhibitors). All procedures were approved by the Regional Committee for Medical and Health Research Ethics WEST, Medical Faculty, University of Bergen / Haukeland University Hospital (REK no 2014/1915) and carried out in accordance with the code of Ethics of the World Medical Association, Declaration of Helsinki. Before the tests, all participants gave written informed consent to participate in the study. Participants were compensated with 100NOK for participation in this study. (See Table 1 for an overview of the participants).

**Table 1:** Group characteristics.

	N (f)	Age (sd/min/max)	Edu (sd/min/max)	MMS (sd/min/max)	MUSLEN (sd/min/max)
**PD**	19 (6)	65.26 (11.54/40/81)	14.21 (4.01/9/18)	27.79 (1.62/24/30)	12.47 (16.17/0/63)
**HC**	19 (8)	65.47 (12.28/40/84)	15.79 (3.13/9/18)	28.52 (1.26/25/30)	14.84(20.68/0/66)
***p* <**		0.957	0.191	0.127	0.696
**PD Group**		**UPDRS-III** (SD/min/max)	**Symptoms** (SD/min/max)	**Diagnosis** (SD/min/max)	
		19.33 (6.01/11/34)	8.11 (4.4/1/17)	6.22 (3.66/2/15)	

PD: Parkinson’s group. HC: Healthy controls. f: Females. Muslen = years playing an instrument. Other columns: Means (standard deviations / minimum / maximum). Edu: Years of education. MMS: Minimal mental-state test. MUSLEN: Length of playing an instrument. UPDRS-III: Unified Parkinson’s disease rating scale, Part III. Symptoms: Years since first self-perceived symptoms. Diagnosis: Years since diagnosis.

### Stimuli construction

Using Steinberg Cubase 7 (http://www.steinberg.net), we created 10 rhythms in 4/4 time signatures, in two modes (major/minor), each in three different tempi (90, 120 and 150 beats per minute, bpm), making a total of 60 stimuli. Each stimulus consisted of eight repetitions of the rhythm, and the stimuli lasted approx. 22, 16 and 13 seconds for 90, 120 and 150 bpm respectively. Rhythms were created with sampled drum sounds and modes where constructed by alternating between simple two-note piano chords at the 1^st^ beat-position of every bar. Major/minor modes were used to make the stimuli pool a slightly more varied listening experience, to avoid repetition of the exact same sound stimuli, while allowing us to present each rhythm twice at each tempo. No analysis was planned or performed for modes. The three tempi chosen were all well within the boundaries of music (see Introduction for more details on tempo). To make the stimuli more musical (and ecological more valid), a bass synth sound was superimposed on each drum onset, alternating between two tones in the set mode of the stimuli. Although the addition of musical instruments added complexity to the stimuli, and potentially added melodic accents that might have influenced the perception of the rhythms [83, 84], we would argue that the uniformity of the stimuli (i.e., no expressive timing, uniform volume on each sound onset and the uniformity of the alternations) strongly limited these as confounding factors, and preserved the role of onsets as the dominant source of temporal or rhythmic information. For complexity ratings of the rhythmic patterns, we used a model by Jeff Pressing, found in [85] (which, in reference to the point above on melodic accents, explicitly uses alternating notes as examples). In this model, rhythmic complexity theoretically is understood as “cognitive costs incurred in maintaining the metrical framework, motor and cognitive costs in the physical production of the pattern, and cognitive costs in reconciling the pattern’s placement within the metrical framework.” It is this latter cost of reconciliation that is the basis of his complexity measures, where combinations of onset positions in sub-divisions of a bar, are given a score, and the sum of these scores give a one-dimensional score for complexity for the whole phrase. The rhythms in our studies are all based on a 4/4 metric structure, so cognitive cost of maintenance of the metrical framework should be equal between them. Since our studies are passive by design, meaning they were simply listening and not performing the rhythms, they also do not involve physical production costs. The reconciliation cost is therefore–theoretically–an ideal measure of the “cost” or cognitive load of perceptual complexity. Pressing’s model thus allowed us to construct a pool of stimuli by manipulating the sequential onset positions of the auditory events. The ‘scaffolding’ for 10 different rhythms in 4/4 time signatures was produced using Pressings complexity scores, where combinations of onset positions in sub-divisions of a bar are given a score, and the sum of these scores gives a one-dimensional score for complexity for the whole bar. To exemplify, in Table 2, rhythm #1 has four identical bars, each with onset only on the first position. In Pressing’s model this sub-bar is given the value of zero, thus the whole rhythm is given the complexity score of zero. In rhythm #10, the first sub-division has onsets in position 1 and 4, giving a “Pressing-score” of 4.5; the second and third sub-division have onsets only in position 3, with a score of 5; the last sub-division has an onset in the second position, with a score of 7.5. This gives rhythm #10 a total ‘Pressing-score’ of 22 for the whole bar (4.5 + 5 + 5 + 7.5). We chose Pressing’s model because it allows for a pragmatic a priori ranking of stimuli complexity. We would however want to stress that the motivation for this study was to investigate if people with PD and HCs judged complexity in different ways, *not* whether Pressing’s model itself was correct. We will also refrain from comparing this model to other models, although we recognize the vast number of studies and approaches in the field [86–98]. For further details on Pressing’s model, we will refer to the original paper [85] as well as Toussaint’s use of it and his comparison to other models [98].

**Table 2:** Overview over the 10 rhythmic patterns used.

Rhythm													Complexity	Integer ratios
1	X			x			x			x			0	4:4:4:4
2	x			x	x		x	x		x			2	4:2:2:2:2:4
3	x	x			x		x			x			6	2:4:2:4:4
4	x			x	x			x		x			6	4:2:4:2:4
5	x			x		x				x	x		7,5	4:3:5:1:3
6	x		x		x			x		x			14,5	3:3:4:2:4
7	x		x		x			x	x				15	3:3:4:1:5
8	x		x			x		x		x			17	3:4:3:2:4
9	x		x		x			x				x	19,5	3:3:4:4:2
10	x		x		x			x			x		22	3:3:4:3:3

Black boxes on top signifies quarter notes of each 4/4 bar, and x’s signifies sound onsets. The complexity scores according to Jeff Pressing’s model [85] in the right column.

Rhythms #4, 6, 7, 8, 9 and 10 in Table 2 were taken from examples in the literature [98], while the remaining four rhythms were constructed based on Pressing’s model. An example of the stimuli used can be found in the supplementary material, as can an overview of the sub-division values in Pressing’s model (S1 and S2 Figs).

### The online survey

The online survey was programmed on a LAMP-platform (Linux, Apache, MySQL, Php), using HTML and JavaScript, ensuring that it ran on most popular operating systems and browser combinations. Stimuli sound files were compressed to mp3-format (320kbp, 44.1kHz). The participants completed the survey at home, using their own equipment. Due to unfamiliarity in using computers and a computer mouse, three of the participants (one PD and two HCs) performed the listening test under the supervision of the first author by giving oral response to the questions and rating schemes.

Participants filled out an online questionnaire with questions about sex, age, handedness, height, weight, education and musical background. The question on musical background included how often participants listened to music in their everyday, if and for how long the participant had played an instrument or sung, whether they considered themselves music-lovers, and which level of musicianship they perceived they had. Additional questions about time since first self-perceived symptoms and time since diagnosis, use of music in therapy, and medication use were added for the people with PD. As stated above all people with PD included in the study were in medication regimens when undertaking the online survey, and were asked to do the test in a strong ON period of medication. All participants in both groups were right handed.

Participants rated the 60 stimuli, presented in a random order and were given no other instructions than “How complex do you perceive this rhythm to be?”, and “How much do you like this rhythm”. Ratings were done on 11-point Likert-scales (ranging from “Very simple”/”Very complex” and “Not at all”/”A lot” respectively). The survey started and ended with the presentation of four rhythms on one screen, giving the participants an opportunity to get a “feel” for the range of stimuli complexity, but no explicit indication of the different levels of complexity of the four samples were given.

The California Verbal Learning Test-II [78] and Stroop-test [79] were done in separate sessions, simultaneously with Mini Mental Test and UPDRS-III examination [82] for the people with PD. Due to scheduling problems, UPDRS-scores for one patient are missing.

### Analysis

We first compared neuropsychological test-scores between the two groups for baseline purposes. For our main research question, i.e., whether the PD-group ratings of the rhythmic stimuli would be influenced by level of complexity and tempo, we performed a series of planned between-group comparisons of complexity-ratings and likeability- ratings, both across tempi and across the varied complexity of the ten rhythms. Secondly, we compared the relationship between complexity and likeability ratings between the two groups. Thirdly, correlation and regression-analyses were performed to investigate closer the relationships between different clinical and cognitive scores, and complexity ratings.

Statistical analyses were done using SPSS (Version 24.0.0.0 / IBM). Permutation-tests and graphical representations t-tests were done using R version 3.5.2 (The R Foundation for Statistical Computing).

## Results

### Neuropsychological tests

For the California Verbal Learning Test-II (CVLT-II) [78], we concentrated on three measures aimed to assess different memory and learning related processes, as utilized in [68]. The CVLT-II started with the examiner reading out a list of 16 words to be immediately repeated by the participant. This procedure was repeated five times (trials 1–5), and the number of correctly remembered words were scored per trail. The sum score of trials 1–5 is sensitive to general memory impairment, while the sum score of trials 2–5 is a composite measure of memory and learning, and finally the learning slope of trials 1–5 is a measure of encoding ability [68]. The Stroop test measures set-shifting abilities [79], and for this test we calculated the inference score (IG) proposed by Golden (1978). This test has previously been used as a measure of attention [80]. At baseline, no significant between-group results were found for any of the neuropsychological test scores (see Table 3).

**Table 3:** Difference between neurological test-scores.

	PD mean (sd)	HC mean (sd)	*p* =
**CVLT-II (1–5)**	39.89 (11.3)	47.68 (14.83)	0.08
**CVLT-II (2–5)**	34.79 (9.68)	41.79 (13.24)	0.07
**CVLT-II (Slope)**	1.26 (0.61)	1.36 (0.65)	0.48
**Stroop1**	80.89 (18.83)	86.37 (12.68)	0.29
**Stroop2**	55.89 (14.48)	58.05 (12.64)	0.63
**Stroop3**	29.89 (13.09)	29.84 (12.04)	0.99
**Stroop IG**	-2.53 (10.59)	-4.48 (9.72)	0.56

CVLT-II: California Verbal learning test. CVLT-II (1–5) total sum for trials 1–5. CVLT-II (2–5) total sum for trials 2–5. CVLT-II (Slope): Learning slope trials 1–5. Stroop1-3: The three parts of the Stroop test. Stroop IG = Inference score (Golden 1978). Stroop 2,3 and IG scores for one participant in the PD-group is missing (and excluded in that comparison).

### Planned comparisons

A repeated measures-ANOVA, with ratings as a dependent factor, and task (with two levels—ratings for complexity and likeability), rhythm (with ten levels for gradient rhythms) and tempi (three levels—90, 120 and 150bpm) as within-subject factors, and group as between-subject factor was done. Mauchly’s test showing violations of the assumption of sphericity, and Greenhouse-Geisser estimates were used to correct the degrees of freedom. Results at a 95% confidence interval found a significant between-subject effect for group (*F*(1, 36) = 4.112, *p* = .05, ηp2 = 0.103). Significant within-group main effects were found for rhythm (*F*(5.68, 204.38) = 8.49, *p*<.001, ηp2 = 0.19) and tempo (*F*(1.39, 50.22) = 11.14, *p*<.001, ηp2 = 0.24). Significant two-way interactions were found for tempo and group (*F*(1.39, 50.22) = 3.93, *p* = 0.04, ηp2 = 0.10), tempo and rhythm (*F*(7.61, 273.94) = 10.17, *p*<0.001, ηp2 = 0.22), task and rhythm (*F*(6.25, 224.91) = 7.17, *p*<.001, ηp2 = 0.17), task and tempo (*F*(1.39, 49.92) = 23.35, *p*<.001, ηp2 = 0.39). A three-way interaction was found for task, tempo and rhythm (*F*(6.60, 237.47) = 5.61, *p*<.001, ηp2 = 0.13).

Our main analysis was a series of planned, between- and within-group t-tests on tempo and rhythm across the two tasks (i.e., complexity and judgment ratings). Unless otherwise indicated, all t-tests were two-sided, and reported with uncorrected p-values. The exception are the planned comparisons for each individual rhythm, where, because of our strong hypothesis, one-sided t-tests and false-discovery rate (fdr) *q*-values are used.

#### Overall effects of judgment type, and judgment type across tempi

**Fig 1:**
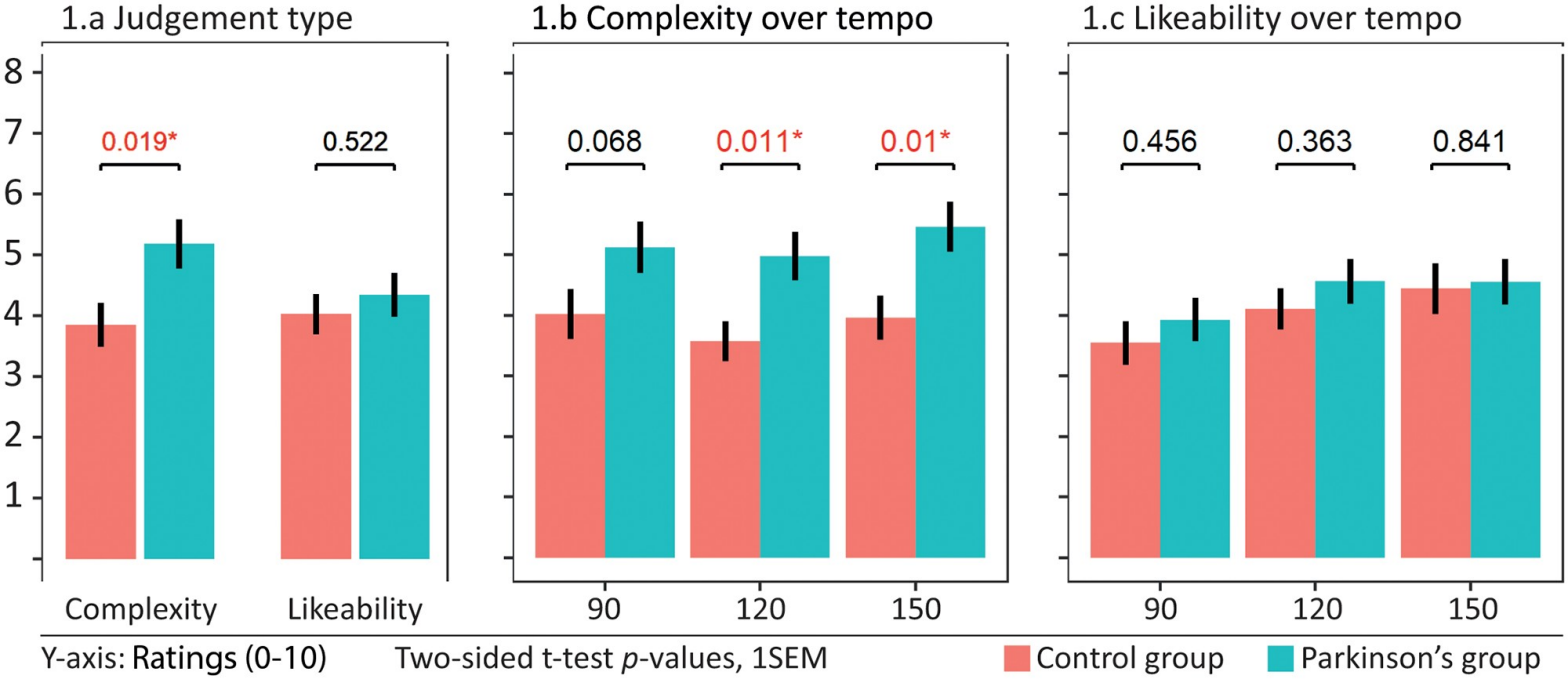
Between-groups differences for complexity and likeability ratings across all rhythms. Two-sided t-tests for between-group differences for overall complexity and likeability ratings (1.a), between-group differences for overall complexity (1.b) and likeability ratings (1.c) for three different tempi. Error bars represent 1 standard error of the mean (SEM).

For complexity ratings (across all rhythms and tempi), there was a significant between-group difference (*p* = .019, Cohen’s *d* = 0.80), where the Parkinson’s group as expected judged the rhythms more complex than the healthy controls. For likeability ratings (across all rhythms and tempi) there were no between-group difference (*p* = 0.522, *d* = 0.21) (Fig 1a).

For group differences for complexity ratings per tempo, we found significant between-group differences for the two fastest tempi (120bpm *p* = 0.011, *d* = 0.87 and 150bpm *p* = .01, *d* = 0.88), but not for the lowest tempo (90bpm, *p* = 0.068, *d* = 0.61). For both groups there were within-group differences between the two fastest tempi (120<150bpm, PD: *p* = 0.001 / HC: *p*<.001), while only the healthy control group showed a significant difference between 90bpm and 120bpm (*p* = 0.008). Neither group showed significant differences between 90 and 150bpm. For both groups, the medium tempo (120bpm) demonstrated lower complexity ratings in absolute numbers than the other two tempi (Fig 1b).

For likeability ratings per tempo there were no significant between-group differences for any tempo, but both groups showed significant within-group differences between 90/120bpm (PD: *p*<0.001 / HC: *p* = 0.005) and 90/150bpm (PD: *p*<0.001 / HC: *p* = 0.025), but not between 120/150bpm. Both groups judged the lowest tempo the least likeable. The control group judged 150bpm slightly more likeable than 120bpm in absolute numbers (Fig 1c).

#### Differences between all ten rhythms, across tempi

**Fig 2:**
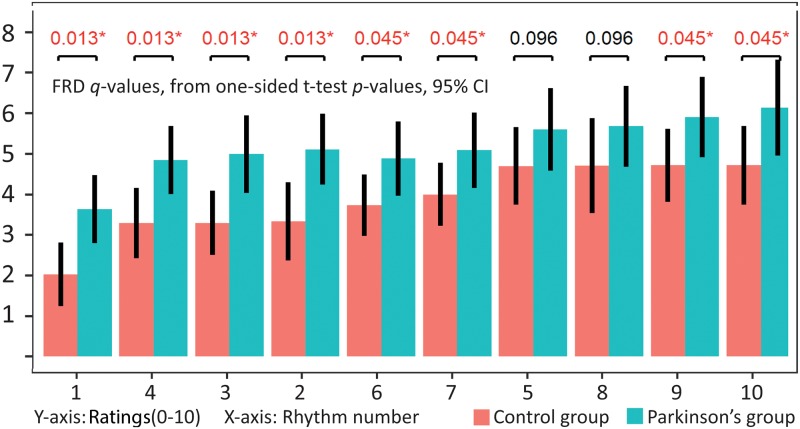
Between-group differences for complexity ratings. Between-groups fdr *q*-values from one-sided t-test *p*-values for averaged complexity ratings for each of the 10 individual rhythms. Error bars indicate 95% confidence interval. Y-axis indicates ratings of complexity (0–10) while X-axis are the 10 rhythms, ordered by increasing complexity ratings in the control group.

Based on previous findings in the literature, our *a priori* prediction was that the PD-group would give higher complexity ratings for the rhythmic stimuli. While our other t-tests (above) were done as two-sided tests, the comparison of the ten individual rhythms where performed as one-sided t-tests for the complexity judgments, using fdr-correction for multiple comparison (*q*-values). Comparisons of the likeability ratings were done with two-sided t-tests, since we did not have any strong hypothesis about these ratings. Including all 10 rhythms across all tempi, we found significant between-group differences for 8 of the 10 rhythms (Fig 2). For likeability ratings we did not find any significant difference for any of the ten rhythms. Testing for tempi, only one rhythm showed a significant group difference for complexity ratings at the slowest tempo (90bpm, #4 *q* = 0.040). For 120bpm, six of the rhythms were significantly different (#1–4, #6 and #10). For 150bpm eight rhythms were significantly different (#1–4,6,7,9,10). For likeability ratings, no significant differences were found between any of the rhythms at individual tempi.

#### Rating of complexity and likeability

To test how well the two groups rated the ten rhythms for complexity, compared to our a priori ranking and to test how the two groups compared to each other (summed across all tempi), a linear-trend analysis was performed. A significant Mantel-Haenszel linear-by-linear association between Pressing-rank (Table 2) and group averaged rankings were found both for the PD-group (*M*^2^ = 5.33, *df* = 1, *p* = 0.021) and the HC-group (*M*^2^ = 7.42, *df* = 1, *p* = 0.006). A significant between-group association was also found (*M*^2^ = 6.85, *df* = 1, *p* = 0.009). Range normalization–a way of scaling data with different ranges into the same unit interval–allows a more direct comparison of the relative intervals between scores across the 10 rhythms. We range normalized the “pressing-scores” and the subjective scores (by group) into a unit interval ranging from 0 to 1 and repeated the above analysis. Again a significant association between the Pressing-rank and complexity ratings in both groups was found (PD: *M*^2^ = 5.02, *df* = 1, *p* = 0.025 / HC: *M*^2^ = 5.68, *df* = 1, *p* = 0.017), with a between-group association (*M*^2^ = 8.12, *df* = 1, *p* = 0.004). For likeability ratings, neither group showed any association with Pressing’s ranking of complexity, for neither rank ordered or range-normalized data, which we also would not assume since Pressing’s scores are for complexity, but the analysis showed a significant between-group association for both rank ordered (*M*^2^ = 6.03, *df* = 1, *p* = 0.014) and range-normalized data (*M*^2^ = 4.16, *df* = 1, *p* = 0.041).

#### Relationship between complexity and likeability-scores

**Fig 3:**
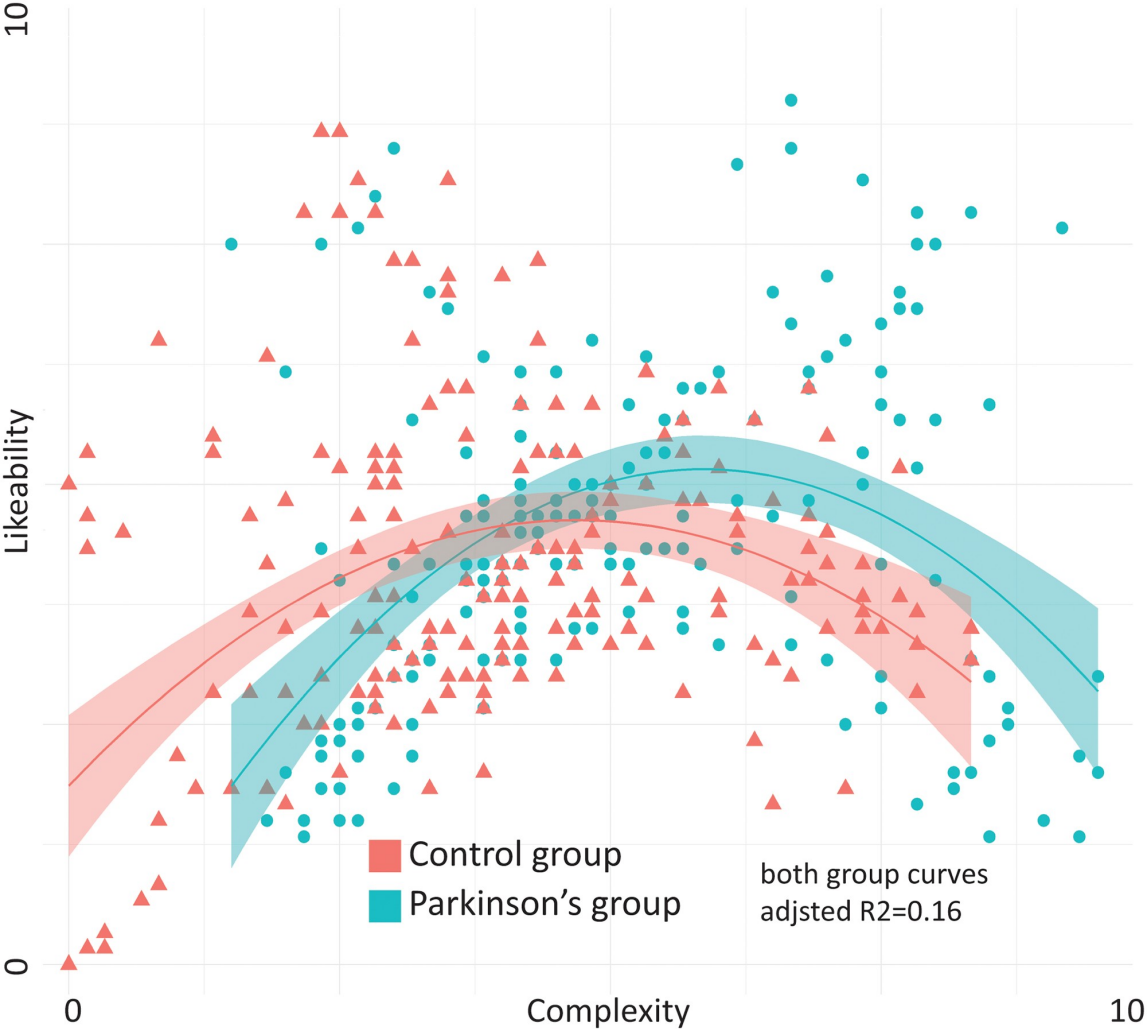
Likeability vs complexity-scores. Quadratic curve-fit for both groups shows inverted U-curves (Wundt-curves) for the relationship between likeability scores (y-axis) and complexity scores (x-axis).

Examining the relationship between complexity ratings and likeability ratings to check for a hypothesized inverted U-curve, we did regression analysis of average complexity and likeability ratings for each participants in each group. In both groups a quadratic model yielded poor model fit with an adjusted R^2^ = 0.16 for both groups. They were however both significant (F(2,187) = 18.89, *p*<0.001 for the PD-group and F(2,187) = 18.96, *p*<0.001 for the healthy controls). There was no statistical difference between the curves for the two groups, but as Fig 3 shows, there shift towards higher complexity rating can be seen in the PD-group.

#### Correlations between subject judgment-scores and other factors

Pearson’s correlation tests were carried out within the two groups to check for correlations between overall complexity and likeability ratings respectively, with age, sex, education, years of playing an instrument, neuropsychological test scores (CVLT-II-scores (1–5, 2–5 and slope), Stroop-tests (inference score), and MMS scores), and disease length (years since diagnosis) and UPDRS-II scores for the PD-group. No significant correlations where found (all *p*>0.087 and *p*>0.103 for the healthy controls and the PD-group respectively).

### Regression analyses

A forward stepwise linear regression analyses was carried out to examine whether results in neuropsychological test-scores could predict complexity ratings (using complexity ratings as dependent and CVLT-II, Stroop, and MMS-scores as independent variables), but yielded no significant predictor variables.

Forward stepwise binary logistic regression analysis was carried out to examine whether group could be predicted from complexity ratings, years of playing an instrument, CVLT-II (1–5,2–5,slope), Stroop (IG), and MMS-scores. Stroop-scores from on participant was missing, but the analysis excluding this participant showed that Stroop was not a significant predictor variable, and this variable was removed so that the analysis could be done with all participants. A model including CVLT(2–5) and complexity ratings was significant (χ2(2) = 10.83, *p*<0.004), and better than complexity ratings alone (χ2(1) = 5.95, *p*<0.015), and explained 33% (Nagelkerke R^2^ = 0.331) of the variance and correctly classified group-belonging with 68.4% (in comparison with 19% and 63.2% respectively for the simpler model that just included complexity ratings).

To assess the reliability of the observed effect in the above analysis, we randomly permuted the group labels 1000 times and ran logistic regressions based on the model including the CVLT(2–5) and the complexity ratings. Permuting the data like this generated a null distribution of the complete data-set, and if the observed effect were higher than the last 5% if the null distribution (expressed as *p*-values), this would be a strong indicator that the observed effect is reliable. We thus compared both the observed accuracy (68.4%) and the R^2^ (33%) with the permuted results, and found that the observed R^2^ value was significant (*p*<0.006) while the accuracy of classifying group-belonging was not, although close to trending (*p*<0.085). Running the permutation on the reduced model, including only the complexity ratings, found that both the observed accuracy of classifying group-belonging (63.2%) and the R^2^ (19%) to be significant (*p*<0.023 and *p*<0.045 respectively).

## Discussion

Previous research is not conclusive about whether the level of complexity differentially affects the ability for discrimination, i.e., whether–in a binary fashion–people with PD have greater difficulties discriminating simple than complex rhythms relative to healthy controls (a “beat-based impairment” [15, 44]) or whether this impairment is of a more general nature, as suggested in a recent study [45]. The “beat-based impairment” hypothesis indicates that people with PD, compared to healthy controls and, in a binary fashion, are more impaired on discrimination on strongly metric (simple or isochronous) rhythms than more complex rhythms. If this assumption was correct, we would expect to find that the statistical difference between the two groups would be significantly larger for the rhythms judged least compared to those judged more complex. Furthermore, should this “beat-based impairment” not be exclusively binary, but nonetheless still depend on the relative level of complexity of the rhythms, one would assume that the group differences would inversely scale with the level of complexity. As our results show, we found that the PD-group gave similar overall higher complexity-ratings both for simple and complex rhythms, and that the group difference did not scale with complexity. Our findings are thus not consistent with a “beat-based impairment” [15, 44], but points to a more overall, generalized impairment [45], independent of complexity levels, at least in the pure subjective, perceptual judgment of rhythm in a musical context.

The results were also modulated by tempo, a variable not investigated in previous research on rhythm perception in Parkinson’s disease. When taking tempo into consideration, our analysis showed no significant overall between-group difference at the lowest tempo (90bpm), while the group-difference remained intact for the two faster tempi. Across the ten rhythms, our analysis of the more varied complexities gave a more fine grained understanding, particularly of the effect of tempo. The number of rhythms showing significant differences increased with tempo, from one at the slowest tempo, six at the medium tempo and eight at the fastest tempo. This indicates an effect of the interaction between complexity and tempo. Interestingly, complexity ratings were not associated with tempo–in fact both the slowest and the fastest tempi were judged as overall more complex than the middle tempo of 120bpm (or 2Hz) in both groups. This tempo is viewed as a key spontaneous tempo of human locomotion [50], particularly salient when walking to music [51] and has also been used to investigate perception of rhythm in relation to complexity [57]. As a strong auditory-motor area coupling is a dominant explanatory model for beat perception [16, 17, 52, 53], music at a tempo close to that of natural gait or dancing might be experienced as less complex than slower and faster tempi. The effect of tempo on perceived complexity should be investigated further in future studies, perhaps also by including tempi matched to the natural gait of the participants.

Both groups showed a significant association with the a priori Pressing-ranking, and we note that this indicates that Pressing’s model holds a potential as a simple and useful tool for stimuli construction for more varied complexities. More telling is the strong association between the two groups in their ranking of the complexities of the ten rhythms. We believe this is an indication that the PD-group in fact has a *preserved* ability for *relative* complexity judgments, with a consistent elevated baseline for complexity judgments, again inconsistent with the simple dichotomy that underlies the idea of a potential “beat-based deficit”. The tempo-dependent judgments and the elevated baseline-level in PD for complexity in rhythm should be investigated in other modalities using for example temporal visual and tactile paradigms, to see if this effect is confined to the auditory domain alone, or whether it is an expression of a domain-independent cognitive impairment, since impairments of judgment of complexity could simply be the result of a general cognitive impairment in the PD-group.

The dissimilarity between complexity and likeability ratings, where the PD-group gave higher ratings for all complexity ratings, but similar ratings for likeability ratings relative to the healthy controls, indicate that judgment of complexity and judgment of likeability are two cognitively different operations. Both groups showed hints of an inverted U-curve previously found in the literature, where medium complexities are preferred to too simple or too complex rhythms. This further points to a preserved, although displaced, relationship between complexity and likeability in PD.

It is tempting to speculate that this indicates that PD-pathology affects the neural underpinnings for complexity-judgments but not the neural underpinnings for likeability-judgments, perhaps due to differential involvement of basal ganglia-loops for the different operations. To this, we will add that as judgment of complexity asked the participant to address one particular quality of the stimuli, this could be seen as a more cognitively demanding task than judging for likeability, and in addition, the quality we asked to be judged (complexity) spoke directly to well known temporal processing impairments in Parkinson’s disease [13, 48].

However, the opposite might be true, that complexity-judgments are not so cognitively demanding, and instead contingent on motor-abilities, as indicated in a recent study that shows that rhythmic and musical skills in people with PD influence gait benefits of rhythmic auditory cueing. We also do not know enough about the cognitive and neuronal underpinnings of likeability judgments to turn this speculation into a claim. This is however a topic that would be highly interesting to investigate further, also in other pathologies, as it relates more to affective traits than motor and temporal traits.

The all-round lack of between-group differences in likeability ratings indicate that the two groups did not use the *scales* differently, i.e., that the method of investigation itself did not disfavor the PD-group. We would suggest that this similarity in judgment of likeability therefore can serve as a control task for future research in perception of rhythmic complexity in Parkinson’s disease, and potentially also in other pathologies.

UPDRS-III is a clinical assessment of level of all motor related problems, and has previously been found to correlate with complexity discrimination [45], indicating that rhythm discrimination and motor symptoms could be related. In the current study, we did not find however correlation between complexity judgments and UPDRS-III scores. A previous study [15] indicated that rhythm perception impairment in Parkinson’s disease has certain specific qualities, i.e., that rhythmic processing in Parkinson’s disease could be used as predictors for other cognitive operations. We did not find correlations between complexity ratings and any of the neuropsychological tests (CVLT-II, Stroop and MMS). Lack of correlations might be due to the low number of participants in this study. We also used different tests, so our lack of significant correlations does not refute the findings in those papers. However, as our regression analysis showed, a combination of specific cognitive tests scores and complexity ratings correctly classified group belonging with almost 70% accuracy, and we firmly believe further research into the specificity of the relation between various rhythmic tasks and cognitive testing in other domains (such as learning, memory and attention) is a fruitful way forward to better understand and identify the cognitive impact of Parkinson’s disease, using musical rhythms as research tools.

### Limitations

While most studies use “pure tone” (sinusoid synthesized sounds) stimuli, we opted for more ecological valid stimuli using sampled drums and mode-dependent alternating piano and bass sounds. This increased the spectral complexity in the stimuli and could have influenced the results. It could also have altered the perception of rhythmic accents, although Pressing’s model does not lend weight to these and on the contrary explicitly uses alternating notes in the examples. We also used more repetitions than in previous studies. In the study by Grahn [44] the stimuli are not repeated but played only twice with a pause between them. It is possible that such a single repetition design in our study would have yielded other results.

Using an internet-based survey has some challenges in that we are moving outside the controlled situation of the laboratory. We had no control over the quality of the equipment the participants were using, and we cannot control the level of attention, distraction or other factors that might influence the responses of the participants. One important limitation to our study is for example that we did not measure our participants ability for synchronization abilities, and have no way of knowing whether the used covert or overt tapping or bodily movements as an aid to assess the complexity of the rhythms.

On the other hand, allowing the participants to perform the test in their own, known environment, might in fact increase the ecological validity of their judgments, in line with Honing [99]. We would argue that performing these tests in the confines of the laboratory *increase* the cognitive load, because the participants must then also address the novelty of the situation (being in a new and unknown, often confined space). Using their own equipment (known headphones and setting their own volume) also makes the sound characteristics more “familiar”, easing the task. That the participants can do the test when they “feel up to it”, also means that the participants are probably rested and more focused than attending to an otherwise enforced time. Some of the participants also reported this sentiment. In addition, the practical demands of getting to and from labs put additional demands on people with Parkinson’s disease, in both a physical and psychological sense. We will therefore argue that using an internet-based tool, with highly motivated participants is a valid research tool in rhythm and music perception studies, also in PD.

Another potential explanation for the elevated baseline of complexity in people with PD could be that they were aware that they took part in a larger study on rhythm perception and projected their own phenomenological experience of having PD into their answers: a belief that they *should* perceive it as more complex than what they really did, although this could also be argued the other way: That they would defiantly try to disprove this assumption. Mental states such as depression are well documented as frequent comorbidities in PD, and this could therefore have influenced the ratings. We did not perform any tests for depression or quality of life in the current study, and this would be a useful addition in future studies.

## Conclusion

We found overall higher, but not complexity dependent, subjective ratings of complexity of musical rhythms in the PD-group. Examining the effect of tempo as well as using a more varied spectrum of complexity shows a more detailed picture than previous dichotomous simple/complex conditions. The overall impairment in rhythm perception interacts with tempo. The results in our study indicates that people with PD have a *preserved* ability for *relative* complexity discrimination, but with an elevated baseline-level for complexity judgments. Furthermore, the dissimilarity between judgments of complexity and judgments of likeability between the two groups, indicates different neuronal underpinnings for the two different types of judgment, where PD-pathology affects one but not the other. Future studies should investigate the dissociation by comparing self-selected, familiar music, pure tone and metronome stimuli across complexity- and tempo-variations, as well as explore the effect of repeated exposure in stimuli presentation. More knowledge of these phenomena could potentially inform intervention related designs through a more informed selection of music used in therapy.

## Supporting information

S1 Fig Stimuli construction, rhythm # 6. (TIF)

S2 Fig Pressing’s model, values of subdivisions. Pressing’s model of cognitive rhythmic complexity. Calculations for values on sub-bars based on position. Scores on each sub-bar is added up to give a total score for the whole phrase (64).
(TIF)

S1 File Complete data set in .XLS format. (XLSX)

S2 File Online questionnaire in Norwegian (original) and English (translated). (DOCX)
