# Supplementary figures and images for: Subjective judgments of rhythmic complexity in Parkinson’s disease: Higher baseline, preserved relative ability, and modulated by tempo
Source: PLoS One. 2019 Sep 3;14(9):e0221752. doi: 10.1371/journal.pone.0221752 (PMC6719828; doi:10.1371/journal.pone.0221752)

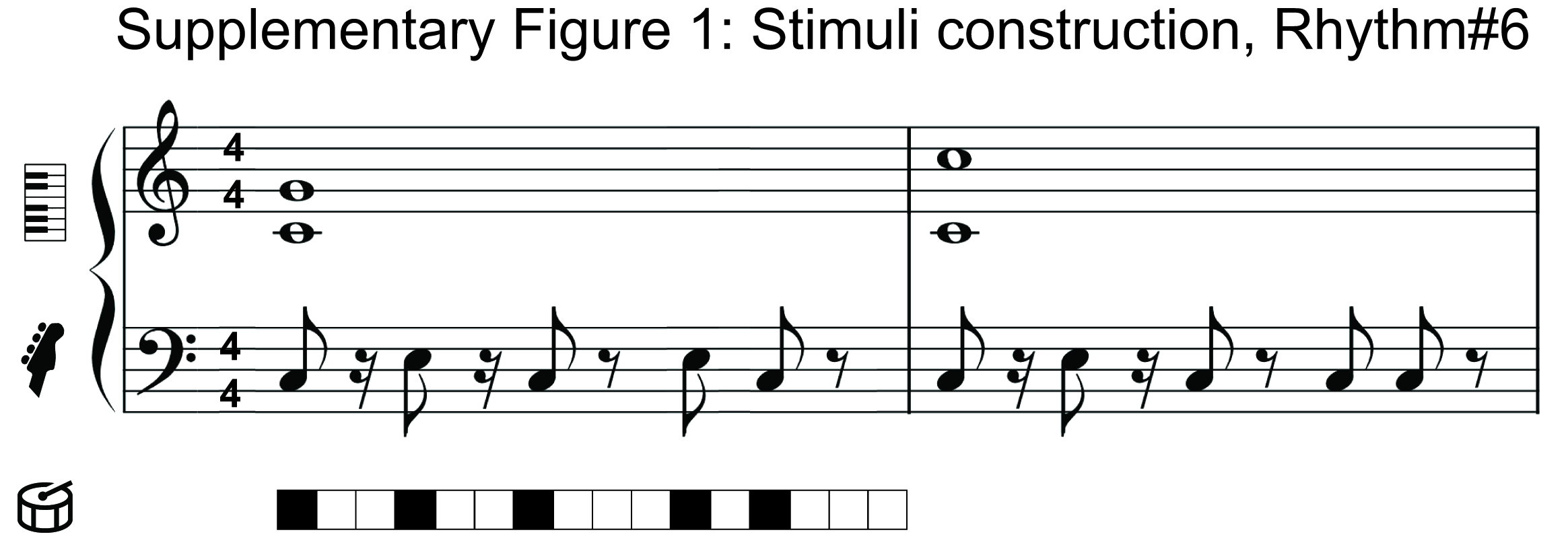

Supplement: S1 Fig — (TIF) [file pone.0221752.s001.tif]

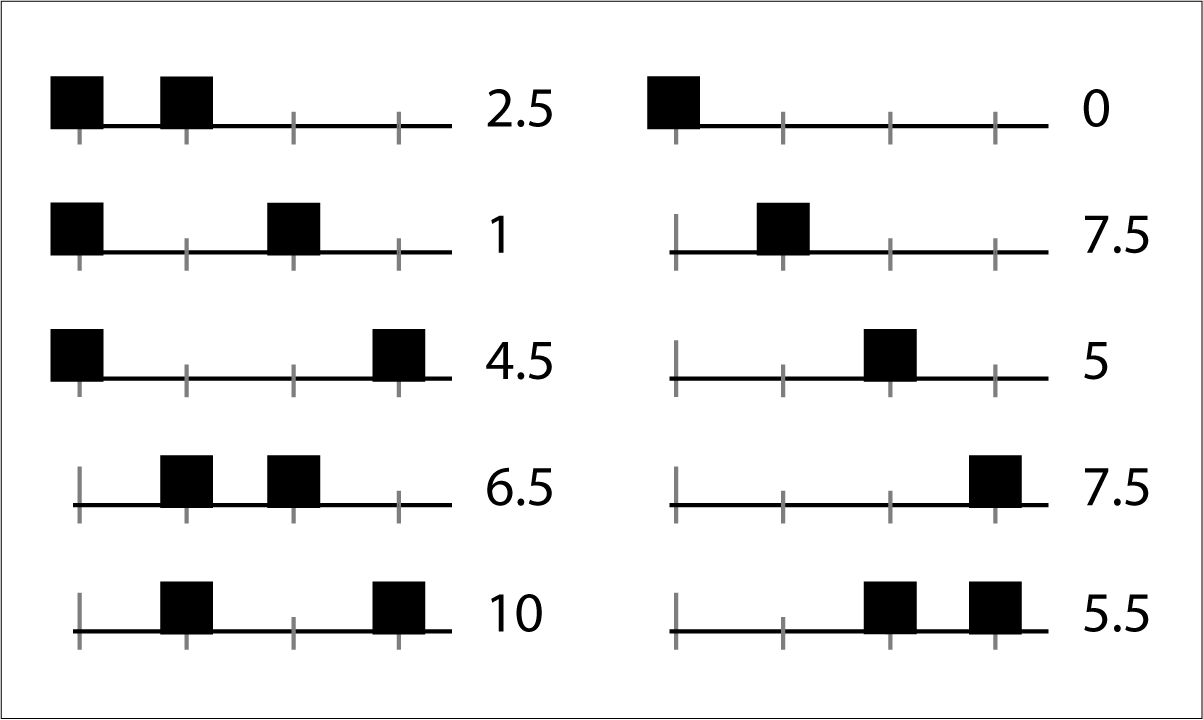

Supplement: S2 Fig — Pressing’s model of cognitive rhythmic complexity. Calculations for values on sub-bars based on position. Scores on each sub-bar is added up to give a total score for the whole phrase (64). (TIF) [file pone.0221752.s002.tif]
