# Supplementary material for: Subjective judgments of rhythmic complexity in Parkinson’s disease: Higher baseline, preserved relative ability, and modulated by tempo
Source: PLoS One. 2019 Sep 3;14(9):e0221752. doi: 10.1371/journal.pone.0221752 (PMC6719828; doi:10.1371/journal.pone.0221752)
Supplement: S2 File — (DOCX) [file pone.0221752.s004.docx]

**SPØRRESKJEMA:**

Kjønn: (Mann / Kvinne)

Hånd: (Høyre / Venstre / Begge)

Høreapparat: (Høyre / Venstre / Begge)

Fødselsår: (ÅÅÅÅ)

Høyde: (i cm)

Vekt: (i kg)

Utdanning: (grunnskole 9 / videregående 12 / høyere utdanning 15+ / hovedfag, master, doktorgrad 21+)

Arbeid: (Ikke Yrkesaktiv / Yrkesaktiv < 50% / Yrkesaktiv > 50% / Pensjonert men aktiv / Pensjonert, ikke aktiv /

Hvilke type jobb / studie har du/hadde du? (Fritekst)

Jeg spiller eller har spilt følgende instrument(er): (Strykeinstrument / Gitar / Sang / Treblåser / Trommer, slagverk / Piano / Messinginstrument / Andre strengeinstrumenter / Annen perkusjon / Keyboard, piano)

Hvor mange år har du spilt et instrument? (AAAA)

Skriv litt om hvor lenge du har spilt de forskjellige instrumentene du har spilt lengst, hvor mye du øver/øvde pr. uke, og hvilket år du eventuelt sluttet å spille. (Fritekst)

Jeg deltar i dag i følgende musikalske aktiviteter: (Live musikk / Synger i kor / Lærer et instrument / Synger hjemme og i dusjen / Spiller i band/gruppe / Musikaler / Dirigerer / Karaoke/ Skriver musikk / Spiller i et lite orkester / Spiller i et stort orkester / DJ'ing / Former for musikkterapi / Spiller instrument hjemme / Annet)

Har du annen informasjon som burde vært med? Deltar du i andre musikalske aktiviteter eller har informasjon om din musikalske bakgrunn som du syns burde vært med kan du skrive det her. (Free text)

Jeg betrakter meg selv som: (Ikke-musiker / Musikkelsker men ikke-musiker / Amatør-musiker / Semi-profesjonell musiker / Profesjonell musiker)

Jeg har også deltatt eller deltar fremdeles aktivt i følgende aktiviteter: ( Drama, Teater ,Skuespill / Dansing, Bevegelse / Idrett, annen fysisk trening og aktivitet)

Kan du kort beskrive disse andre aktivitene? Hvor lenge du har holdt på, når du eventuelt stoppet med disse aktivitene og annen informasjon som du mener er relevant. (Fritekst)

Hvor ofte lytter du til musikk? (Aldri / Av og til / 1-2 dager i uken / 3 - 4 dager i uken / 5 - 6 dager i uken / Hver dag)

Kryss av for de språkene du snakker flytende: (Norsk / Engelsk / Tysk / Fransk / Spansk / Dansk / Svensk / Finsk / Arabisk / Tegnspråk / Russisk / Kinesisk)

Er det andre språk du snakker som ikke finnes på listen her? (Fritekst)

**QUESTIONAIRE:**

Sex: (Male / Female)

Handedness: (Right / Left / Both)

Hearing aid: (Right / Left / Both)

Year of birth: (YYYY)

Height: (in cm)

Weight: (in kg)

Education: (Primary 9 / Highschool 12 / Higher education 15+ / master, PhD 21+)

Work: Not working / Working < 50% / Working > 50% / Retired, but active / Retired, but not active)

Which type of work / study did you/do you do? (Free text)

I play or have played the following instruments: (Bowed instruments / Guitar / Song / Wood / Drums / Piano / Horns / Other stringed instruments / Other percussion / Keyboard, piano)

How many years have you played an instrument actively? (YYYY)

Write briefly about how long you have played the different instruments, how much you rehearse(d) every week and, if applicable, which year you stopped playing. (Free text)

I currently participate in the following musical activities: (Live music / Sings in a choir / Learning an instrument / Sing at home and in the shower / Play in a band or group / Musicals / Directing / Karaoke / Write music / Plays in a small ensemble / Plays in a large orchestra / DJ / Forms of music therapy / Play an instrument at home / Other)

Do you have other information you think should be included? Are you participating in other musical activities or have information about your musical background that you think should be mentioned please write it here. (Free text)

I consider myself: (Non-musician / Musicloving non-musician / Amateur musician / Semi-professional musician / Professional musician).

I am / have also participated in the following actitivites: ( Drama, Teater ,Skuespill / Dansing, Bevegelse / Idrett, annen fysisk trening og aktivitet

Can you briefly describe these activities? How long you did them, when, if applicable, you stopped these and other information you feel is relevant. (Free text)

How often do you listen to music? (Never / Sometimes / 1-2 days a week / 3 - 4 days a week / 5 - 6 days a week / Every day)

Indicate which languages you speak fluently: (Norwegian / English / German / French / Spanish / Danish / Swedish / Finish / Arabic / Sign language / Russion / Chinese)

Are there other languages you speak that are no listed? (Fritekst)
